# Identification of genetic variation in the swine toll-like receptors and development of a porcine TLR genotyping array

**DOI:** 10.1186/s12711-016-0206-0

**Published:** 2016-03-31

**Authors:** Alex Clop, Abe Huisman, Pieter van As, Abdoallah Sharaf, Sophia Derdak, Armand Sanchez

**Affiliations:** Center for Research in Agricultural Genomics (CRAG) Consorci CSIC-IRTA-UAB-UB, Campus UAB, 08193 Bellaterra, Catalonia Spain; Hypor BV, Villa ‘de Körver’, Spoorstraat 69, 5831 CK Boxmeer, The Netherlands; Hendrix-Genetics, Villa ‘de Körver’, Spoorstraat 69, 5831 CK Boxmeer, The Netherlands; Ain Shams University, Cairo, Egypt; Centre Nacional d’Anàlisi Genòmica CNAG, Baldiri Reixac 4, 08028 Barcelona, Catalonia Spain; Departament de Ciència Animal i dels Aliments, Facultat de Veterinària, Universitat Autònoma de Barcelona, 08193 Bellaterra, Catalonia Spain

## Abstract

**Background:**

Toll-like receptors (TLR) are crucial in innate immunity for the recognition of a broad range of microbial pathogens and are expressed in multiple cell types. There are 10 *TLR* genes described in the pig genome.

**Results:**

With a twofold objective i.e. to catalogue genetic variants in porcine *TLR* genes and develop a genotyping array for genetic association studies on immune-related traits, we combined targeted sub-genome enrichment and high-throughput sequencing to sequence the 10 porcine *TLR* genes in 266 pigs from 10 breeds and wild boars using a DNA-pooling strategy. We identified 306 single nucleotide variants across the 10 *TLR* and 11 populations, 87 of which were novel. One hundred and forty-seven positions i.e. six stop-gains and 141 non-synonymous substitutions were predicted to alter the protein sequence. Three positions were unique to a single breed with alternative allele frequencies equal to or higher than 0.5. We designed a genotyping array for future applications in genetic association studies, with a selection of 126 variants based on their predicted impact on protein sequence. Since *TLR4*, *TLR7* and *TLR9* were underrepresented in this selection, we also included three variants that were located in the 3′UTR of these genes. We tested the array by genotyping 214 of the 266 sequenced pigs. We found that 93 variants that involved the 10 *TLR* genes were polymorphic in these animals. Twelve of these variants were novel. Furthermore, seven known variants that are associated with immune-related phenotypes are present on the array and can thus be used to test such associations in additional populations.

**Conclusions:**

We identified genetic variations that potentially have an impact on the protein sequence of porcine TLR. A genotyping array with 80 non-synonymous, 10 synonymous and three 3′UTR polymorphisms in the 10 *TLR* genes is now available for association studies in swine populations with measures on immune-related traits.

**Electronic supplementary material:**

The online version of this article (doi:10.1186/s12711-016-0206-0) contains supplementary material, which is available to authorized users.

## Findings

Toll-like receptors (TLR) are a family of innate immunity receptors, which are expressed in many cell types, including macrophages, dendritic cells, keratinocytes or even sperm cells. Each TLR recognizes a specific range of microbial pathogens and informs the cell to initiate an immune response. While cell-surface TLR (TLR1, TLR2, TLR4, TLR5, TLR6 and TLR10) recognize non-nucleic acid molecules, intracellular TLR (TLR3, TLR7, TLR8 and TLR9) detect nucleic acids. TLR are relatively large proteins. In swine, the sizes of cell surface and intra-cellular TLR proteins range from 785 to 856 and from 905 to 1050 amino acids, respectively (see Additional file 1). Cell-surface TLR are highly polymorphic, especially in the ectodomains that recognize the pathogens, which allows the organism to broaden the catalogue of molecules that it can recognize. In the pig, 10 *TLR* genes (*TLR1* to *TLR10*) have been described and unambiguously mapped to seven genomic regions on chromosomes (chr) 1, 8, 10, 13, 15 and X. *TLR1*, *TLR6* and *TLR10* cluster within a 56-kb interval on chr 8 and *TLR7* and *TLR8* map within a 62-kb region on chr X (see Additional file 1). Genetic and functional approaches have linked several porcine missense single nucleotide polymorphisms (SNPs) that are located within *TLR* genes to immune traits. *TLR1*, *TLR5* and *TLR6* are related to antibody responses after vaccination against *Erysipelothrix rhusiopathiae* or *Actinobacillus pleuropneumoniae*, [1]. *TLR2* is associated with the incidence of pneumonia caused by *Mycoplasma hyopneumoniae* [2]. *TLR5* is associated with the expression of *IL*-*2*, *IL*-*10* and *TLR5*, itself, in peripheral blood mononuclear cells (PBMC), which suggests the presence of a regulatory variant near *TLR5* [3]. Reporter assays have also linked missense SNPs that are located within *TLR2* and *TLR5* to a differential reactivity to *Salmonella enterica* [4] and within *TLR3* to different responses to stimulation by poly(A:C), a synthetic acid that emulates viral infection [5]. Among the 10 porcine *TLR* genes, *TLR4* has by far the largest number of reported genotype:phenotype associations. Missense SNPs within *TLR4* are linked to the expression of *IFNG*, *TNFA*, *IL*-*2*, *IL*-*4* and *IL*-*6* in PBMC as well as to the presence of lesions in the lung [6] and the expression of *TLR2*, *TLR4* itself, *TNFA* and *IL*-*1β* upon lipopolysaccharide stimulation in pulmonary alveolar macrophages [7]. Moreover, porcine *TLR4* is also associated with *Salmonella typhimurium* fecal shedding [8]. In spite of all these reports, the impacts of TLR on most of the health conditions that are relevant to pig breeding remain unexplored.

Currently, targeted sub-genome enrichment in combination with high-throughput sequencing is enhancing research in the field of genetics and, particularly, the identification of genetic variation. We captured and sequenced a sub-exome that consisted of 10 taste receptors, 191 genes from the appetite-reward pathways and the TLR from pools of genomic DNA (gDNA). Here, we describe the identification of genetic variation in the porcine *TLR* genes. We hypothesized that variations that alter the protein sequences of TLR may shape innate immunity and affect disease resistance. The aim of our study was twofold, i.e. (1) to map coding variants in the porcine *TLR* genes and predict their allelic frequencies in several pig populations and their impact on protein sequence; and (2) based on this information, to develop a genotyping array with a set of variants that tag the 10 *TLR* genes for future applications in association studies for immune-related traits.

To select the target regions, we considered the 49 non-redundant exons from the 22 transcripts annotated for the 10 porcine *TLR* genes (www.ensembl.org), including the coding sequences and untranslated regions (UTR). Each *TLR* has one single reference RefSeq (http://www.ncbi.nlm.nih.gov/books/NBK21091) transcript. RefSeq transcripts include 30 of the 49 exons and display the longest coding sequences, which fully encompass the coding sequences of the alternative transcripts. In addition, most of the alternative transcripts share an identical coding sequence and protein product and only differ in the UTR. Overall, the 22 transcripts span 32,278 base pairs (bp) of exonic sequence, of which 26,706 are coding sequences and 5572 are UTR (see Additional file 1). For the design of Agilent’s SureSelect Target Enrichment baits, 31 genomic positions that fully encompass all *TLR* exons (coding sequences and UTR) of the 22 transcripts were retrieved via Ensembl’s Biomart [9] (see Additional file 2).

Genomic DNA (gDNA) samples from 266 pigs belonging to 10 breeds (Large White, Landrace, Pietrain, Duroc, Iberian, Majorcan Black, Bazna, Mangalitza, Meishan and Vietnamese) and wild boars were combined into 14 per-breed pools (see Additional file 3) using semi-equal amounts of gDNA that was quantified using a NanoDrop™ spectrophotometer. Meishan and Vietnamese samples were pooled together in an Asian pool. Specialized professionals from each institution that provided animal material obtained all blood samples and tissues following standard routine monitoring procedures and guidelines. No animal experiment was performed within this research.

Genomic DNA pools were subjected to target enrichment and library preparation according to Agilent’s SureSelect protocol for Illumina multiplexed paired-end sequencing. A detailed description of all the methods is available in Additional file 4. The libraries were then sequenced on two sequencing lanes using an Illumina HiSeq 2000 sequencer, which produced 2 × 100 bp read pairs, following the manufacturer’s instructions (see Additional file 4). Reads were mapped to the porcine reference genome (Sscrofa10.2) with the GEM toolkit [10] and BFAST read aligner [11]. Only the reads that were unambiguously mapped to a unique genomic location were kept for further analysis. GATK 3.1 [12] was used for variant calling (see Additional file 4). Functional predictions were added using snpEff [13] based on the Sscrofa10.2.69 database, which classifies variants according to their impact on protein sequence as High (H), Moderate (M), Low (L) or Modifier variants (see Additional file 5). M variants were further classified as deleterious (Mdel) or tolerated (Mtol) according to SIFT scores (see Additional file 3) using snpSift [14] and the Ensembl’s Variant Effect Predictor (VEP) tool [15]. Variants were considered novel if they were not annotated in the porcine dbSNP version 138 using the VEP tool [15]. The proportion of reads that carried each allele was used to estimate the frequency of the alternative allele (pAAF).

**Fig. 1:**
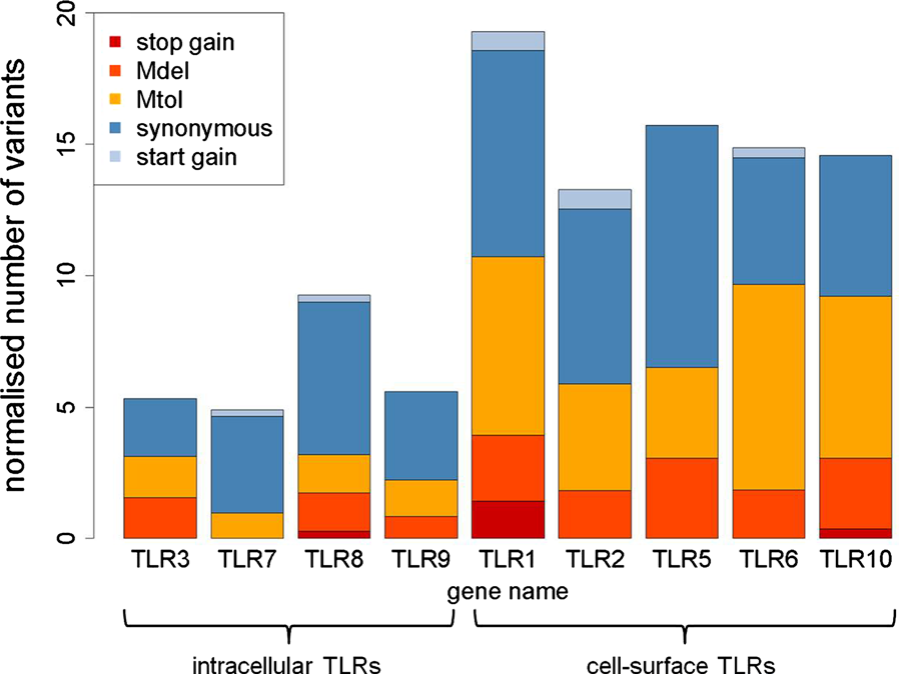
Normalized number of variants for each *TLR* gene and variant type. The normalized number of variants indicates the number of variants for every 1000 of exonic sequence. *TLR4* is not included because of poor sequencing quality

**Fig. 2:**
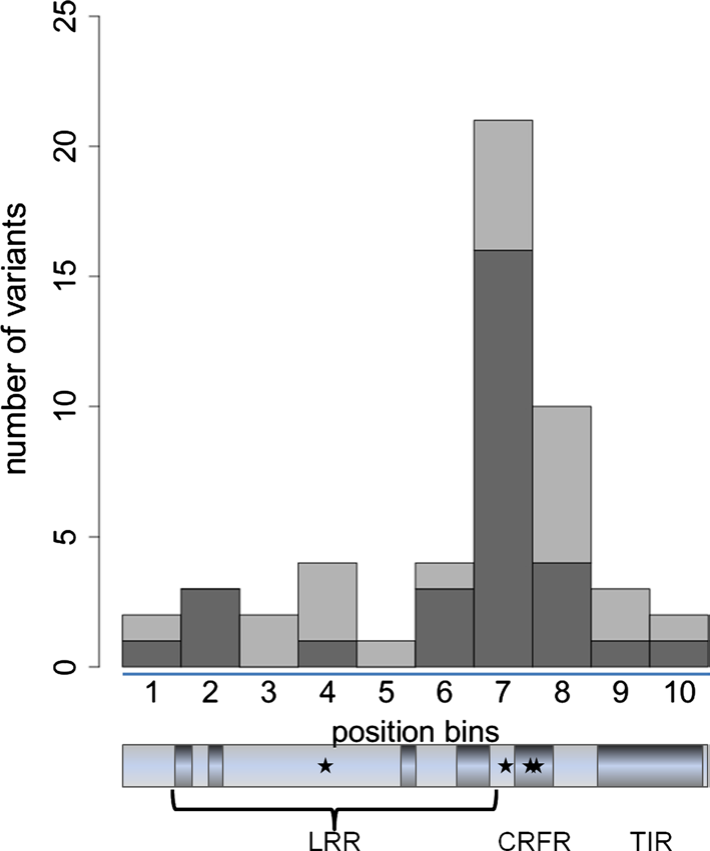
Variant distribution along the porcine TLR1 protein. *Dark grey vertical bars* indicate the number of stop-gain and non-synonymous coding variants. *Light grey vertical bars* indicate the number of synonymous coding variants. Each position bin includes 10 % (79.6) of the TLR1 amino acids. The *dark grey horizontal boxes* show the leucine-rich repeats (LRR), the cysteine-rich flanking region (CRFR) and the toll/interleukin-1 receptor (TIR) domains of the TLR1 protein. *Black stars* represent the four stop-gains identified in *TLR1*

The mean depth for each pool ranged from 3498 to 4794 uniquely mapped sequence reads per bp position (depth) (see Additional file 3). By considering all the pools together, 29,163 bp (90.3 % of the initial target) of the *TLR* exonic sequences were covered with a depth greater than 1000. This threshold was set in order to accurately calculate pAAF for each breed-pool. Only *TLR4* was poorly sequenced with only 41 % of its exons sequenced above that depth (see Additional file 1). Capture baits could be designed for the complete *TLR4* gene but some segments (exon 1, exon 2 and the proximal portion of exon 3) displayed perfect homology to the unmapped genomic contig JH118734.1 that harbors ENSSSCG00000024231, a TLR paralog predicted by Ensembl’s genebuild pipeline. Since the corresponding reads could not be uniquely mapped, they were excluded from further analysis. After read mapping, 306 single nucleotide variants (SNV) were called, among which 87 were not annotated in the porcine dbSNP (version 143) and were thus considered novel SNV (Table 1) and (see Additional file 6). Six SNV (in *TLR1*, *TLR8* and *TLR10*) were predicted to create a premature stop-codon and thus their impact was classified as high (Table 1). One hundred and forty-one non-synonymous SNV were classified as moderate impact variants (45 Mdel and 96 Mtol) (Table 1). The remaining 159 SNV, including 152 synonymous and seven start codon gains (Table 1) were classified as low impact variants. As already described in humans [16], cell-surface porcine TLR had a larger number of variants that included stop-gain and non-synonymous variants (ranging from 13.3 in *TLR2* to 19.3 per kb of exonic sequence in *TLR1*) than intracellular TLR for which the number of variants ranged from 4.9 in *TLR7* to 9.3 in *TLR8* (Table 1 and Fig. 1). Premature stop codons that abolish the toll/interleukin-1 receptor (TIR) domain, which is responsible for signal transduction, will generate TLR that fail to recognize the pathogen-associated molecular patterns on the cells. Nonetheless, these stop-gain variants in cell-surface *TLR* genes are relatively common in humans and it is generally considered that they are tolerated because of functional redundancy between various TLR [16]. In agreement to previous studies in swine [17], *TLR1* had the highest level of variability with 54 SNV that did not have a uniform distribution i.e. 24 (17 protein-changing and seven silent) SNV were located between residues 500 and 565 within the leucine-rich repeat ectodomain responsible for binding pathogen ligands (Fig. 2).

**Table 1:** Number of variants identified per gene and predicted effect on protein sequence

Gene name	High impact	Moderate impact		Low impact		Total
	Stop gain	Mdel	Mtol	Synonymous	Start gain	
*TLR1*	4	7	19	22	2	54
*TLR2*	0	5	11	18	2	36
*TLR3*	0	5	5	7	0	17
*TLR4*	0	0	1	7	0	8
*TLR5*	0	8	9	24	0	41
*TLR6*	0	5	21	13	1	40
*TLR7*	0	0	4	15	1	20
*TLR8*	1	5	5	20	1	32
*TLR9*	0	3	5	12	0	20
*TLR10*	1	7	16	14	0	38
total	6	45	96	152	7	306

Mdel, variants predicted to have a moderate impact on protein sequence by snpEff and to be deleterious by SIFT; Mtol, variants predicted to have a moderate impact by snpEff and tolerated by SIFT

When all the variants were considered across the 11 pig populations, the Asian and Landrace pools displayed the largest levels of genetic diversity whereas it was lowest for the Majorcan Black pool (Table 2). However, these data should be taken with caution because the number of animals varied within each breed-pool. Still these results are not surprising, since the Asian pool included two breeds (Meishan and Vietnamese) and thus, was expected to harbor more genetic diversity, and also Asian and European *Sus scrofa* split around 0.8–1.6 million years ago and are in fact considered as two different subspecies [18]. Since the reference pig genome sequence is derived from a western breed (Duroc) animal, more differences are expected when comparing Asian pig sequences to this reference sequence. In addition, three SNV were present in only one breed and with a pAAF higher than 0.5 in that breed. Two of these breed-specific variants, including a Mdel in *TLR8*, were detected in the Asian pool (Table 2) and (see Additional file 6).

**Table 2:** Number of variants identified for each breed

Variant type	BA	MA	DU	PI	LW	LA	IB	MB	WB	AS
Stop-gain	0	0	2	1	2	1	0	1	1	1
Mdel	16	14	13	18	12	23	6	3	11	19 (1)
Mtol	41	26	41	42	54	56	33	13	34	51
Synonymous	51	46 (1)	68	78	80	72	44	26	49	88 (1)
Start-gain	2	0	2	1	3	5	3	3	1	2
Total	110	86	126	140	151	157	86	46	96	161

When appropriate, the number of breed-specific variants with a pAAF higher than 0.5 is indicated between brackets

*BA* Bazna, *MA* Mangalitza, *DU* Duroc, *PI* Pietrain, *LW* Large White, *LA* Landrace, *IB* Iberian, *MB* Majorcan Black, *WB* wild boar, *AS* Asian

In addition to cataloguing genetic variation in porcine *TLR* genes, an equally important goal of our study was to develop a genotyping array with polymorphisms that tag the 10 *TLR* genes for future association studies on immune-related traits in the swine. For this purpose, we used the OpenArray^®^ genotyping technology (Life Technologies), which accommodates 128 SNV assays (see Additional file 4), and we designed 126 assays that targeted the 10 *TLR* genes and two that targeted the *SRY* gene (c.135C>G and c.593G>C) [19] for sex determination. Due to limitations in the design that are related to the sequence context, including melting temperature and presence of repeat elements or polymorphisms near the target, we could not assay all the variants that altered TLR proteins. First, we designed assays for all the SNV that were predicted to alter protein sequence (six H, 45 Mdel and 96 Mtol). This yielded 113 successful assays for the 10 *TLR*. To increase the number of assays, we also included 10 SNV that were predicted to be silent. After this first step, we found that *TLR4*, *TLR7* and *TLR9* were poorly represented in the array. Hence, we also included one 3′UTR variant for each of these genes. Then, the array was tested by genotyping 214 of the sequenced animals, instead of all 266 pigs because the amount of DNA was not sufficient to genotype the other 52 animals. Genotyping was performed in a QuantStudioTM 12 K Flex Real-Time PCR System (Life Technologies) and the results were analysed by using both Taqman Genotyper version 1.3 and Symphoni Suite software (Life Technologies) (see Additional file 4). Three of the *TLR* SNV did not yield high-quality genotypes. For 30 *TLR* SNV, the alternative allele was not detected, but since the frequency of most of these, including all the H SNV, were low (i.e. 24 had a pAAF lower than 0.01), some may represent real polymorphisms that were present in ungenotyped animals. For 93 *TLR* SNV, the alternative allele was detected for at least one animal, including 16 Mdel, 64 Mtol, 10 L and three 3′UTR variants (Table 3) and (see Additional file 7). Among these, 12 are novel variants and 81 were previously reported by other groups. Moreover, seven of the 81 already annotated SNV are associated to different immune-related traits as discussed below. Although *TLR4* is the most poorly represented gene on the array, it displays three SNV (one Mtol, one synonymous and one 3′UTR), which should, at least partially, tag the gene in genetic association studies. Each sample had on average 21 SNV for which the alternative allele (both in the heterozygous and homozygous states) was detected. In agreement with the findings based on pool sequencing, the largest number of alternative alleles was found for the Meishan animals. In contrast, the Majorcan Black pigs were the least divergent when compared to the reference genome. The 18 rare variants that had allelic frequencies lower than 0.01 were uniformly distributed (one to three variants per animal) across 27 pigs and the breed distribution was in agreement with the sequencing data (data not shown). The frequencies of the alternative allele that were estimated based on genotyping data and the pAAF were highly correlated (Pearson r^2^ = 0.88). To the best of our knowledge, only *TLR1*, 2, 3, 4, 5 and 6 have been genetically or functionally associated to immune related traits in pigs. Some of the missense variants that are described in the previous reports are present on our genotyping array. More specifically, the following variants were included on the array: rs326791928 and rs321053450 in *TLR1*, rs81210417, rs81218850 and rs81218851 in *TLR5*, and rs322825361 in *TLR6* which were associated to antibody response after vaccination against bacteria [1] and rs81218811 in *TLR2* which was linked to the incidence of pneumonia [2]. Moreover, rs81218851 in *TLR5* was also associated to the mRNA expression of *IL*-*2*, *IL*-*10* and itself [3]. This new array will contribute to better understand the genetic impact of these variants and other porcine *TLR* variants on a broader range of immunological traits. This genotyping array is available, by contacting the corresponding author, for collaborative efforts to perform genetic association analyses in swine populations with records on immune-related traits such as infectious disease resistance or auto-immune conditions.

**Table 3:** Number of confirmed polymorphisms from the genotyping array per gene and per predicted effect

Gene name	Mdel	Mtol	Synonymous	3′UTR	Total
*TLR1*	1	8	0	0	9 (19)
*TLR2*	1	8	0	0	9 (13)
*TLR3*	1	5	4	0	10 (13)
*TLR4*	0	1	1	1	3 (3)
*TLR5*	5	9	0	0	14 (14)
*TLR6*	0	14	0	0	14 (20)
*TLR7*	0	3	2	1	6 (7)
*TLR8*	4	2	0	0	6 (10)
*TLR9*	0	4	3	1	8 (10)
*TLR10*	4	10	0	0	14 (17)
Total	16	64	10	3	93 (126)

The number of variants that were originally chosen for genotyping is indicated between brackets

## Additional files

10.1186/s12711-016-0206-0 Genomic information from the 49 *TLR* exons that are annotated in the genome Sscrofa10.2.69 database. Columns A and B (general information) supply general information on the *TLR* gene related to sense of transcription (phase +1: sense; −1: antisense), number of transcripts, protein ID and length. Detailed information on exons order and length of coding sequence and UTR is only supplied for RefSeq exons. RefSeq transcripts are highlighted in bold in column “Ensembl transcript ID”.
10.1186/s12711-016-0206-0 Target *TLR* genomic regions. The coordinates correspond to the Sscrofa10.2 genome assembly. The column “portion without sequence information (bp)” indicates the number of bp that were sequenced to a depth of less than 1000. The column “cds and UTR content” indicates whether that genomic portion correspond to coding sequence or/and to UTR. The column “cause of missing sequence information” indicates the reason for lack of sequence information for the corresponding segment. There are two legends: “missing capture baits” indicate that Agilent capture baits that cover this segment could not be designed; “homologous region in the swine genome” indicates that although the baits were designed and the segment was sequenced, the corresponding sequence reads could not be unambiguously mapped to that region.
10.1186/s12711-016-0206-0 Number of gDNA samples included in each library pool and the corresponding sequencing read depth at each position (DP). Large White, Landrace, Duroc, Pietrain samples were merged in two library pools each according to their commercial origin. The numbers between brackets indicate the number of animals in each of the pools for these four breeds.
10.1186/s12711-016-0206-0 Supplementary methods.
10.1186/s12711-016-0206-0 Variant types according to their predicted effect and impact on protein sequence based on SnpEff.
10.1186/s12711-016-0206-0 List of the 306 *TLR* variants identified in this study. Breed specificity indicates the variants that are present in only that breed and at a pAAF higher than 0.5. In this column, the number between brackets indicates the pAAF in the breed with the variant allele.
10.1186/s12711-016-0206-0 List of the 93 polymorphisms present on the genotyping array. Description: The column “alternative allele frequency” shows the allele frequency of the alternative allele as calculated from the individual’s genotypes.
