# Supplementary material for: Identification of genetic variation in the swine toll-like receptors and development of a porcine TLR genotyping array
Source: Genet Sel Evol. 2016 Mar 31;48:28. doi: 10.1186/s12711-016-0206-0 (PMC4818456; doi:10.1186/s12711-016-0206-0)
Supplement: Supplementary file 4 — 10.1186/s12711-016-0206-0 Supplementary methods. [file 12711_2016_206_MOESM4_ESM.docx]

**Supplementary Methods**

**Samples.** gDNA from 266 pigs belonging to 11 breeds (Large White, Landrace, Pietrain, Duroc, Iberian, Majorcan Black, Bazna, Mangalitza, wild boar, Meishan and Vietnamese) was extracted from blood or solid tissues using a standard phenol-chloroform organic extraction protocol or the Charge Switch gDNA Micro Tissue kit (Invitrogen). The Meishan and Vietnamese samples were merged into a single Asian pool.

**Target enrichment and high-throughput sequencing.** The 14 gDNA pools were subjected to genome target enrichment and library preparation following Agilent’s SureSelect protocol for Illumina paired-end sequencing. Briefly, three μg of gDNA were sheared on a Covaris™ E220 instrument. Fragment size (150-300bp) and yield were confirmed on Agilent 2100 Bioanalyzer 1000 chips. Fragmented DNA was end-repaired, adenylated and ligated to Agilent paired-end adapters. The DNA with adapter-modified ends was PCR amplified (six cycles, Herculase II fusion DNA polymerase). PCR product size and yield were determined on an Agilent 2100 Bioanalyzer DNA 1000 assay and hybridized to the genomic capture probes for 24hrs at 65ºC (Applied Biosystems 2720 Thermal Cycler). The hybridization mix was washed using magnetic beads (Dynabeads MyOne Streptavidin T1, Life Technologies). Indexed tags were added by PCR (16 cycles) using 6bp SureSelectXT indexes for Illumina. Library size and concentration were measured on an Agilent 2100 Bioanalyzer 1000 assay.

The libraries were pooled and sequenced in two lanes of a flow cell in an Illumina HiSeq 2000 instrument following the manufacturer’s protocol, with paired end run of 2x101bp. Image analysis, base calling and quality scoring were processed using Illumina’s Real Time Analysis (RTA 1.13.48) software. FASTQ sequence files were generated using CASAVA software (Illumina).

**Read mapping and variant calling.** Reads were hard trimmed from the end of the read up to the first base with a quality of at least 10 and reads with a length of at least 40nt were mapped to the pig reference genome (Sscrofa10.2) <http://hgdownload.cse.ucsc.edu/goldenPath/susScr3/bigZips/susScr3.fa.gz>). Reads were mapped first with the GEM toolkit [1] allowing up to four mismatches. Unmapped reads were then aligned using BFAST [2] with less stringent settings. Further, we manually removed the variants that clustered in regions with high variant density as these have a strong potential to be false variant calls caused by misaligned reads. Alignment (.bam) files containing only properly paired, uniquely mapped reads without technical duplicates were used for variant calling: Each pool was processed separately. The ploidy of the pool was calculated as twice the sample size in the pool and input as an extra parameter for GATK 3.1 UnifiedGenotyper [3]. For variant calling, read numbers were down-sampled to 1,000 per position.

Variant calls were performed individually for each pool, and then merged into a common multi-sample .vcf file using GATK CombineVariants [3]. The absence of some variants in certain pools was confirmed in a second round of single pool variant calling restricted to the overall list of variant positions in the merged .vcf file. Subsequently, results were merged again. Functional annotations were added using snpEff [4] with the Sscrofa10.2.69 database, and variants were classified according to their predicted impact as High (H), Moderate (M), Low (L) and Modifier. Porcine dbSNP version 138 and porcine SIFT scores and deleteriousness prediction were annotated using snpSift [5] and the Ensembl Variant Effect Predictor (VEP) online tool (<http://www.ensembl.org/Tools/VEP>). M variants were further classified as deleterious (Mdel) or tolerated (Mtol) according to SIFT. TLR genes and the original target region were annotated using vcftools [6]. The number of mapped reads carrying each allele at variant positions in the merged common .vcf file were annotated using GATK Variant Annotator [3]. We used the proportion of reads carrying each allele to calculate the frequency of the alternative allele (pAAF).

**Identification of breed-specific variants.** We searched for allelic variants that were uniquely present in one breed and at a pAAF ≥ 0.5 as these could be considered potentially breed-specific.

**OpenArray design, genotyping and variant validation.** In order to validate the variants with the most likely impact on TLR protein sequence, and to develop a genotyping solution to be employed in future association studies, we developed a genotyping array containing 126 TLR variants detected in the sequencing experiment. We designed the TaqMan® probes for the OpenArray® Real-Time technology for Genotyping (Life Technologies) using the online Custom Assay Design tool. This design requires the absence of DNA variants in the 2 bp vicinity of the target variant, and there are some constrains on the probe’s melting temperature. As a consequence, we could not design assays for all the variants.

The genotyping was performed in a QuantStudioTM 12K Flex Real-Time PCR System (Life Technologies).

250 ng of gDNA and master mix were loaded to the OpenArray plates using the AccuFillTM robotic system (Life Technologies), filled with an immersion fluid and sealed. OpenArray plates were genotyped according to the manufacturer’s protocol. Genotype analysis was performed using both Taqman Genotyper version 1.3 and Symphoni Suite software (Life Technologies).

References:

1. Marco-Sola S, Sammeth M, Guigó R, Ribeca P. The GEM mapper: fast, accurate and versatile alignment by filtration. Nat Methods 2012, 9.

2. Homer N, Merriman B, Nelson SF. BFAST: An alignment tool for large scale genome resequencing. PLoS One 2009, 4:e7767.

3. DePristo M a, Banks E, Poplin R, Garimella K V, Maguire JR, Hartl C, Philippakis A a, del Angel G, Rivas M a, Hanna M, McKenna A, Fennell TJ, Kernytsky AM, Sivachenko AY, Cibulskis K, Gabriel SB, Altshuler D, Daly MJ: A framework for variation discovery and genotyping using next-generation DNA sequencing data. Nat Genet 2011, 43:491–498.

4. Cingolani P, Platts A, Wang LL, Coon M, Nguyen T, Wang L, Land SJ, Ruden DM, Lu X. A program for annotating and predicting the effects of single nucleotide polymorphisms, SnpEff: SNPs in the genome of Drosophila melanogaster strain w1118; iso-2; iso-3. Fly (Austin) 2012, 6:1–13.

5. Cingolani P, Patel VM, Coon M, Nguyen T, Land SJ, Ruden DM, Lu X. Using Drosophila melanogaster as a model for genotoxic chemical mutational studies with a new program, SnpSift. Front Genet 2012, 3:1–9.

6. Danecek P, Auton A, Abecasis G, Albers C a., Banks E, DePristo M a., Handsaker RE, Lunter G, Marth GT, Sherry ST, McVean G, Durbin R. The variant call format and VCFtools. Bioinformatics 2011, 27:2156–2158.
